# FGF23 is correlated with iron status but not with inflammation and decreases after iron supplementation: a supplementation study

**DOI:** 10.1186/1687-9856-2012-27

**Published:** 2012-10-26

**Authors:** Vickie Braithwaite, Andrew M Prentice, Conor Doherty, Ann Prentice

**Affiliations:** 1 MRC Human Nutrition Research, Elsie Widdowson Laboratory, Cambridge CB1 9NL, United Kingdom; 2 MRC International Nutrition Group, London School of Hygiene and Tropical Medicine, London, WC1E 7HT, UK; 3 MRC Keneba, Keneba, West Kiang, The Gambia

**Keywords:** Fibroblast growth factor-23, Iron status, Inflammation, Africa, Iron supplementation

## Abstract

**Background:**

Recent studies have described relationships between iron status and fibroblast growth factor-23 (FGF23) but the possible confounding effects of inflammation on iron status have not been considered. The aims of this study were a) to consider a relationship between FGF23 and inflammation b) to identify relationships between iron status and FGF23 whilst correcting for inflammation and c) to assess the relationship between changes in FGF23 and iron status after supplementation.

**Study design and methodology:**

Blood samples from an iron supplementation study in children (*n=*79) were collected at baseline and after 3 months supplementation with iron sulphate. The children were from a rural Gambian population where rates of iron deficiency and infection/inflammation are high. This study identified cross-sectional and longitudinal relationships between FGF23, inflammation (C-reactive protein (CRP)) and iron status (ferritin, haemoglobin, and zinc protoporphyrin). CRP ≥ 5 mg/dL was used to indicate inflammation and FGF23 ≥ 125 RU/mL was considered elevated.

**Results:**

FGF23 was not significantly correlated with CRP. At baseline, all markers of iron status were significantly correlated with FGF23. Ferritin was the strongest independent inverse predictor of FGF23 in subjects with and without elevated CRP (coefficient (SE)): All subjects=−0.57 (0.12), R^2^=22.3%, *P*≤0.0001; subjects with CRP < 5 mg/dL=−0.89 (0.14), R^2^=38.9%, *P*≤0.0001. FGF23 was elevated in 28% of children at baseline and 16% post supplementation (*P*=0.1). Improved iron status was associated with a decrease in FGF23 concentration in univariate (ferritin =−0.41 (0.11), R^2^=14.1%, *P*=0.0004; haemoglobin=−2.22 (0.64), R^2^=12.5%, *P*=0.0008; zinc protoporphyrin=1.12 (0.26), R^2^=18.6%, *P*≤0.0001) and multivariate analysis (R^2^=33.1%; ferritin=−0.36 (0.10), *P*=0.0007, haemoglobin = −1.83 (0.61), *P*=0.004, zinc protoporphyrin=0.62 (0.26), *P*=0.02).

**Conclusions:**

Iron status rather than inflammation is a negative predictor of plasma FGF23 concentration. Improvements in iron status following iron supplementation are associated with a significant decrease in FGF23 concentration.

## Background

Fibroblast growth factor-23 (FGF23) is a bone derived, phosphate regulating hormone which is often elevated in genetic hypophosphataemic disorders [1] and in chronic kidney disease [2]. Recent studies have identified relationships between FGF23 and various markers of iron status. These include an inverse correlation between FGF23 and serum iron (Fe) [3,4], haemoglobin (Hb) [5], and ferritin (Ferr) [6]. However each of these markers of iron status is affected by the acute phase response and consequently they have limited use as markers of nutritional iron status when used in isolation from markers of inflammation [7]. It is plausible that the inverse correlation between markers of iron status and FGF23 [3-6] is the result of confounding by the inflammatory response. The potential role of the inflammatory response in FGF23 pathways has not yet been considered [8].

The aims of this study were a) to consider a relationship between FGF23 and inflammation b) to identify relationships between iron and FGF23 whilst correcting for inflammation and c) to assess the change of FGF23 after supplementation with iron. The study used data from children in a rural Gambian population where dietary calcium intakes are low, rates of iron deficiency [9] and infection are high, and where a wide range of FGF23 concentrations have been reported [5].

## Methods

### Subjects and study information

A subset of samples (*n*=79) from a cohort of children (*n*=821) who participated in a non-controlled iron supplementation study designed to assess the variability of response to supplementation during the malaria season were included in this study. The children in the cohort were recruited from the local community in West Kiang, The Gambia and were aged between 0.5-7.0 y. Baseline measurements and start of supplementation were conducted in August 2004 and post supplementation measurements were conducted in December 2004 [10]. Subjects were given Fe 6x/week for three months in the form of Fe sulphate tablets (Nutriset, Malaunay, France). Daily consumption of the supplement was supervised by trained fieldworkers. The dose given was dependent on age and, if present, the severity of baseline anaemia (<7 g/dL): 25 mg elemental Fe in 76 subjects and 50 mg in 3 severely anaemic subjects. A blood sample and anthropometric data were collected at both time-points. All samples from subjects aged between 4.8–6.0 y with sufficient residual EDTA-plasma stored from samples at the beginning and end of supplementation (*n*=79, 47 female/32 male) were included in this study and analysed for plasma FGF23. The mean (SD) time from measurements at baseline to post supplementation was 118 (2) days.

### Ethical statement

Ethical approval from The Gambian Government/MRC Laboratories Joint Ethics Committee for the original iron supplementation trial (*n=*821) and the subset study (*n*=79) was obtained. Written informed consent from the parents of the children involved were obtained for both the original iron supplementation trial and for any further analyses performed on stored samples.

### Anthropometry

Weight was measured (kg) using a calibrated electronic scale (model HD-314, Tanita B.V., Hoofddorp, The Netherlands). Height was measured (cm) using a portable stadiometer (Leicester Height Measure, Seca GmbH & Co., Hamburg, Germany). Body mass index (BMI) was calculated (kg/m^2^).

### Fasting blood samples

In the original study an overnight fasting, venous blood sample had been collected into lithium-heparin (LiHep) and EDTA coated tubes. Fresh blood had been used to measure Hb by haemoglobinometer (Medonic CA, 530 Oden 16 Parameter System) and zinc protoporphyrin (ZnPP) by haematofluorimeter (Aviv Biomedical Lakewood, NJ). The remainder of the blood had been separated by centrifugation at 4°C and frozen at −70°C, and had been later transported frozen on dry ice to MRC Human Nutrition Research (HNR), Cambridge, UK where it had been stored at −80°C until analysis. Ferr and CRP had been measured in LiHep by Immunoassay (Abbot Laboratories, IL, USA and Dade Dimension®, respectively) [10]. For the purpose of this study, the residual, frozen EDTA samples were analysed for FGF23 using a 2^nd^ generation C-terminal, two-site enzyme-linked immunosorbent assay (Immutopics Inc., Ca, USA). Assay accuracy and precision were monitored using kit controls supplied by the manufacturer.

### Statistical analysis

All statistical analyses were performed using DataDesk 6.1 (Data Description Inc., NY, USA) with the exception of two-tailed Chi-square tests (without Yates’ correction) which were performed using GraphPad QuickCalcs (GraphPad Software, Inc., CA, USA). Normally distributed data are presented as mean (1SD), positively skewed distributions of data are presented as geometric mean (−1SD, +1SD) obtained from the antilog of mean (−1SD, +1SD) of the logged values. Variables with positively skewed distributions were transformed to natural logarithms before further statistical analysis. Paired t-tests were used to assess the significance of change in anthropometric and biochemical variables from baseline and after supplementation.

A regression model was used with log_e_ FGF23 as the independent variable. Age was not significantly correlated with any of the biochemical variables and was therefore not adjusted for in the regression model. CRP ≥ 5 mg/dL was used to indicate the presence of inflammation [11] and an upper-limit of ≥ 125 RU/mL, as defined by the manufacturers, was used for FGF23. Subjects were defined as being anaemic according to WHO guidelines: Hb ≤ 11.0 g/dL for < 4.99 y, and ≤ 11.5 g/dL for 5–12 y [7,11]. Subjects with Ferr < 15 ng/mL were considered low [11]. Selectors for CRP < 5 and ≥ 5 mg/dL were used in regression analysis. Relative change (∆) in variables (X) over time was calculated by: *ΔX* = log_e_X_post_ - log_e_X_pre_. Univariate analyses were conducted to determine the relationships between ∆FGF23 as the dependent variable, with ∆ in Ferr, Hb and ZnPP in turn. Multivariate analysis was conducted to determine the relationship between ∆FGF23 as the dependent variable, and ∆ in the three markers of iron status markers together and results are described as coefficient (SE).

## Results

At baseline the mean age of the subjects was 5.5 (0.3) y, 28% had elevated FGF23, 87% were anaemic, 24% had low Ferr, and 13% had a CRP ≥ 5 mg/dL. After supplementation, the prevalence of elevated FGF23 was 16% (*P*=0.1), the prevalence of anaemia had decreased to 62% (*P=*0.0005), there were no subjects with low Ferr (*P*≤0.0001) and the frequency of subjects with CRP ≥ 5 mg/dL did not significantly change (*P*=1.0).

After supplementation both Hb and Ferr had significantly increased from baseline (Hb (g/dL) from 10.3 (1.3) to 11.3 (1.0) *P*≤0.0001; Ferr (ng/mL) from 26.6 (12.3, 57.6) to 46.9 (28.6, 76.9) *P*≤0.0001). ZnPP (μmol/mol haem) significantly decreased by the end of supplementation (from 61.9 (32.4, 118.3) to 24.7 (15.8, 38.5), *P*≤0.0001). Mean FGF23 concentration had not significantly changed from baseline to after supplementation (from 96.0 (38.4, 240.4) to 82.8 (47.1, 145.7) RU/mL, *P*=0.1) (Table 1); although there was a wide range of changes observed (mean (−1SD, +1SD) change in FGF23 = 16.2 (4.6, 56.9) RU/mL).

**Table 1:** Anthropometric and biochemical variables at baseline and after iron supplementation

**Dependent**	**Baseline**	**After supplementation**	**Paired t-test**
**Variable**	***n= 79***	***n= 79***	**P-Value**
Age (y)	5.48 (0.32)	5.81 (0.32)	-
Sex (F/M)	47/32	47/32	-
Height (cm)	105.6 (6.8)	107.7 (6.5)	≤0.0001
Weight (kg)	15.6 (2.2)	16.3 (2.2)	≤0.0001
BMI (kg/m^2^)	13.9 (1.2)	14.0 (1.2)	0.2
ZnPP (μmol/mol haem)*	61.9 (32.4, 118.3)	24.7 (15.8, 38.5)	≤0.0001
Hb (g/dL)	10.3 (1.3)	11.3 (1.0)	≤0.0001
Ferr (ng/mL)*	26.6 (12.3, 57.6)	46.9 (28.6, 76.9)	≤0.0001
FGF23 (RU/mL)*	96.0 (38.4, 240.4)	82.8 (47.1, 145.7)	0.1
CRP (mg/dL)*	2.45 (1.00, 6.01)	1.99 (0.83, 4.78)	0.1

Data shown as mean (SD) or geometric mean (−1SD, +1SD) for skewed variables (*).

There was no correlation between FGF23 and CRP at baseline (coefficient (SE)=-0.01 (0.11), R^2^=−1.3%, *P*=0.9) or at the end of supplementation (0.11 (0.07), R^2^=1.8%, *P*=0.1).

**Figure 1:**
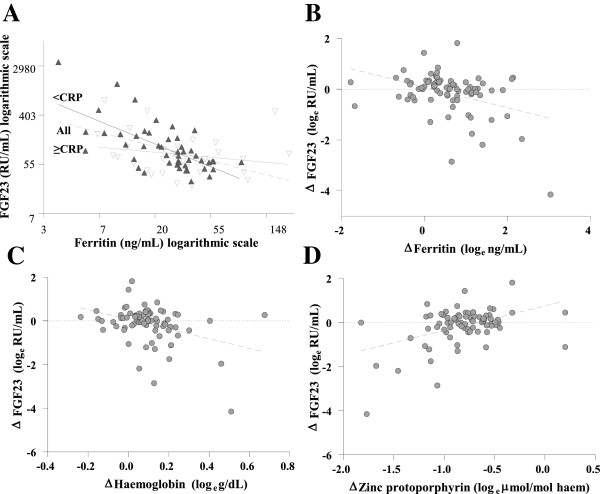
**Cross-sectional and longitudinal relationships between plasma FGF23 with markers of iron status.** Relationship between plasma FGF23 and markers of iron status at baseline (**A**) where subjects are divided by CRP and with change (∆) overtime (**B-D.**) where *ΔX* = log _*e*_*X*_*post*_ − log _*e*_*X*_*pre*_ The equations of the line for **A**: Subjects with CRP<5 mg/dL = ▲: log_e_FGF23 = [7.32 (SE 0.44)] - [0.89 (SE 0.14) (log_e_Ferr)], R^2^=38.9%, *P≤*0.0001. Subjects with CRP ≥ 5 mg/dL = ∇ : log_e_FGF23 = [2.60 (SE 1. 83)] + [0.39 (SE 0.42)(log_e_Ferr)], R^2^=−1.0%, *P=*0.4. All subjects together = : log_e_FGF23 = [6.44 (SE 0.39)] - [0.57 (SE 0.12) (log_e_Ferr)], R^2^=22.3%, *P≤*0.0001. **B**: *Δ*FGF23 = [0.08 (SE 0.11)] − [0.41 (SE 0.11)(*Δ*Ferritin)], R^2^=14.1%, *P=*0.0004. **C**: *Δ*FGF23 = [0.07 (SE 0.11)] − [2.22 (SE 0.64)(*Δ*Haemoglobin)], R^2^=12.5%, *P* =0.0008. **D**: *Δ*FGF23 = [0.76 (SE 0.22)] + [1.12 (SE 0.26)(*Δ*Zinc protoporphyrin)], R^2^=18.6%, *P≤*0.0001.

At baseline, FGF23 was significantly correlated with all markers of iron status. Ferr was the strongest, independent predictor of FGF23 in log_e_-log_e_ models (−0.57 (0.12), R^2^=22.3%, *P*≤0.0001). When excluding subjects with CRP ≥ 5 mg/dL, the relationship between FGF23 and Ferr strengthened (−0.89 (0.14), R^2^=38.9%, *P*≤0.0001) (Figure 1).

The ΔFGF23 was significantly negatively correlated with Δ in markers of iron status in univariate (Ferr = - 0.41 (0.11), R^2^=14.1%, *P *= 0.0004, Hb=−2.22 (0.64), R^2^=12.5%, *P* = 0.0008 and ZnPP = 1.12 (0.26), R^2^ = 18.6%, *P* ≤ 0.0001) analysis. In multivariate analysis, Δ in all three markers of iron status predicted R^2^ = 33.1% of the variation of ΔFGF23 (Ferr = - 0.36 (0.10), *P* = 0.0007, Hb = −1.83 (0.61), *P* = 0.004, ZnPP = 0.62 (0.26), *P* = 0.02).

## Discussion

This study has shown the existence of elevated FGF23, both before and at the end of iron supplementation (28% and 16% respectively) in apparently healthy, although generally undernourished children. This rate is in keeping with previous prevalence findings in Gambian children of similar age [5].

In this study, we have used a combination of iron status markers coupled with CRP, an acute phase marker [11], to provide further evidence of an inverse relationship between FGF23 and iron status.

In keeping with previous reports [9], ~90% of children in this study were anaemic at baseline. Overall Hb concentration increased after supplementation, although ~60% of children were still defined as anaemic [11]. Furthermore, after supplementation the concentration of the other markers of iron status, namely Ferr and ZnPP, changed sufficiently to indicate that the cohort had improved iron status.

Although CRP was not a significant predictor of FGF23, the association between FGF23 and Ferr was further strengthened when children with a raised CRP were removed from the regression model (R^2^=38.9%, *P≤*0.0001). All of the iron status markers were independently correlated with FGF23 at baseline and Ferr was the strongest predictor of FGF23 concentration (R^2^=22.3%, *P≤*0.0001). In addition the decrease in FGF23 concentration was significantly correlated with the improvement of all of iron status markers (both in univariate and multivariate analyses) during the period of iron supplementation. A point to note is that due to the wide range of FGF23 concentrations within subjects the decrease in mean FGF23 concentrations after supplementation compared with baseline was not statistically significant (*P*=0.1).

Although a causal relationship cannot be established, this study shows that FGF23 concentration decreased when iron status improved. This differs from a previous report which described an increase in FGF23 concentration in response of intravenous iron therapy in patients with iron deficiency anaemia and normal baseline FGF23 concentrations [12] but is in keeping with the observation that poor iron status was associated with higher C-FGF23 in Gambian children [5] and in British adults [6]. It has been suggested that low iron may result in an increased rate of proteolytic cleavage of the intact and biologically active hormone to inactive C- and N-terminal fragments. However, recent western-blotting analysis of plasma from Gambian children has not provided any evidence in support of this [13].

In light of this, the more plausible scenario is that iron is involved in the expression and/or secretion of FGF23 rather than having effects on the degradation pathway. This scenario could be further substantiated through the use of the intact FGF23 assay. Unfortunately, the intact FGF23 assay was not available for use in this study.

A limitation of this study is that there was no control group as all subjects were iron supplemented as part of the original study. Therefore, changes seen in FGF23 may not be attributable to the supplementation. Another limitation of this study is that only one marker of inflammation was measured and CRP is a marker of acute rather than chronic inflammation.

## Conclusion

In conclusion this study has demonstrated that FGF23 concentration is not correlated with inflammation but is negatively correlated with markers of iron status. Furthermore an improvement in iron status, associated with iron supplementation in a population with endemic iron deficiency, is associated with a decrease in plasma FGF23.

## Abbreviations

FGF23: Fibroblast growth factor-23; CRP: C-reactive protein; SE: Standard error; Hb: Haemoglobin; Ferr: Ferritin; EDTA: Ethylenediaminetetraacetic acid; Fe: Iron; BMI: Body mass index; ZnPP: Zinc protoporphyrin; MRC HNR: Medical Research Council Human Nutrition Research; LiHep: Lithium-heparin; SD: Standard deviation.

## Competing interests

The authors have no competing interests to declare.

## Authors’ contributions

Study design: VB, AMP, CD, AP. Data analysis: VB, AMP, AP. Data interpretation: VB, AMP, AP. Drafting manuscript: VB, CD, AMP, AP. AP takes responsibility for the integrity of the data analysis. All authors read and approved the final manuscript.
